# Self- and Other-Directed Aggressive Behavior and Safety Measures in an Acute Psychiatric Ward

**DOI:** 10.3390/jcm15208187

**Published:** 2026-10-21

**Authors:** Rosaria Di Lorenzo, Federico Mordenti, Ilaria Rubini, Lisa Capitani, Chiara Biral, Sergio Rovesti, Paola Ferri

**Affiliations:** 1 Department of Mental Health and Drug Abuse, Azienda USL-Modena, 41121 Modena, Italy; 2 Department of Mental Health and Drug Abuse, Azienda USL-Romagna, 48121 Ravenna, Italy; federico.mordenti@auslromagna.it; 3 School of Specialization in Psychiatry, University of Modena and Reggio Emilia, 42122 Reggio Emilia, Italy; 4 Nursing Programme, University of Modena and Reggio Emilia, 41124 Modena, Italy; capitanilisa@gmail.com; 5 Department of Biomedical, Metabolic and Neural Sciences, University of Modena and Reggio Emilia, 41125 Modena, Italy

**Keywords:** aggressive behaviors, acute psychiatric ward, aggression safety measures

## Abstract

**Background**: Aggression in hospital settings, particularly in acute psychiatric wards, is a significant and frequent phenomenon. **Objectives**: This study analyzes self- and other-directed aggressive behavior and highlights risk factors, prevention and management actions. **Methods**: We divided hospitalizations in the Service for Psychiatric Diagnosis and Care (SPDC) of AUSL-Modena over a year based on the presence or absence of aggressive behavior. Socio-demographic and clinical variables, aggression risk assessment, modality of aggression management, Incident Reports, and harmful consequences were analyzed. The data were statistically analyzed. **Results**: The sample consisted of 411 hospitalizations and 370 inpatients, of whom 29.2% (*n* = 108) exhibited aggression: 3.0% (*n* = 11) self-directed, 23.2% (*n* = 86) other-directed, and 3.0% (*n* = 11) both types of aggression. Risk levels were found to be highly predictive of aggressive behavior. Males engaged in other-directed aggressive behavior more frequently than females, who presented more prevalent self-directed aggression (Pearson Chi2 = 13.73; *p* = 0.003). Adolescents more frequently engaged in both self- and other-directed aggression. Other-directed aggressive behavior occurred more frequently in unmarried people (*p* = 0.014), with no children (*p* = 0.019), in involuntary hospitalizations (*p* = 0.000), in longer hospitalizations (*p* = 0.0001), and in acute psychosis (*p* < 0.001), during the first days of hospitalization. The Brøset Violence Checklist showed good discrimination with high sensitivity and negative predictive value. **Conclusions**: Assessing the level of self- and other-directed aggression risk at admission is essential for adopting appropriate preventive and management measures for patient and staff safety in a ward, avoiding coercive measures to contain aggression.

## 1. Introduction

The World Health Organization (WHO) defines aggression and violence as “the intentional use of physical force or power, threat or ability to result in injury, death, psychological harm, deprivation” (WHO 2002) [1]. In 2002, the WHO reported the first world investigation on violence and health “to raise the awareness on violence globally, to make violence preventable, and to highlight the crucial role that public health has to play in addressing its causes and consequences” [1]. Subsequently, in 2008, the WHO launched a report on the prevention of violence and its impact, suggesting that violence should be considered not only a criminal justice issue but also a health issue with social and economic development complications [2].

Aggression can be categorized into other- and self-directed aggression [3], the most extreme form of the latter being suicide, which may represent “the killing of an internalized object” according to an historical psychoanalytic interpretation [4] or a response to unbearable psychological pain [5,6]. Aggression can also be classified into physical and verbal, direct and indirect, defensive and offensive, overt and latent, or proactive and reactive, but various forms of aggression have similar underlying factors associated with aggressive behavior, such as anger (the emotion) and hostile attribution bias [7]. The current theory of cognitive neo-association posits that aggression is a result of associative networks of an emotional state that consists of specific feelings, thoughts, and physical arousal evoked by frustrating events [8]. Anger plays a significant role in the display of different types of aggressive behavior, and anger management can have an impact on reducing aggression, in particular relational aggression [9].

### 1.1. Risk Factors for Aggression

Aggressive behaviors generally have a multifactorial etiology [10,11], arising from the interaction of demographic, clinical, familial, and social factors: male sex [12], younger age [13], a family background of violent behavior [14], a history of childhood abuse [15], alcohol or substance misuse, the presence of psychiatric disorders [16], or brain injuries [17].

However, the literature does not demonstrate a significant correlation between aggression and age, ethnicity, or admission diagnosis [18], whereas a positive association has been observed with substance abuse and a history of violent convictions [19].

Risk factors for aggression in a hospital setting can be divided into three categories:

- Internal risk factors related to the individual (age, gender, lifestyle, psychiatric diagnosis, and medical history).
- Characteristics of the surrounding environment: A psychiatric ward will see overstimulation as a risk factor, due, for example, to a crowded and noisy environment, but the same place could also be a source of understimulation, linked to a lack of activities that leads to boredom. Other triggers include reduced privacy, coupled with a lack of freedom and limited personal space, and spatial organization, such as the arrangement of furniture within the ward [20].
- Variables related to the staff on duty: Staff training and experience; a reduced number of staff members, which influences the frequency of violent incidents; and the gender of staff members, as men are more likely to be involved in physically aggressive incidents [21], while women are more likely to be victims of verbal abuse. Interpersonal factors also play a role, such as the way staff communicate with hospitalized patients [22].

Understanding the various causes and factors that can lead to aggressive behavior is essential to managing aggressive behavior correctly and effectively.

### 1.2. Aggressive and Violent Behavior in Healthcare Settings

Healthcare workers face a 16-fold higher risk of being victims of aggression compared to the general population, and nurses represent the most at-risk category [23]. The highest-risk settings include psychiatric wards, geriatrics, long-term care units, orthopedics and traumatology departments, community nursing, and emergency services or emergency departments [24]. Notably, between 14% and 20% of patients admitted to psychiatric wards commit at least one act of violence during hospitalization [11,25], and approximately 10% of adult patients exhibit either self-directed or other-directed aggressive behavior [26].

During involuntary treatment, lack of insight into the need for care may facilitate aggressive episodes [26,27,28,29]. Nevertheless, the literature highlights that the crime rate among people with psychiatric disorders is comparable to that of the general population [30], and most psychiatric patients do not exhibit aggressive behavior [31].

### 1.3. Aggressive Behavior and Psychiatric Disorders

Some psychiatric disorders can predispose individuals to both self- and other-directed aggressive behavior, which may represent a trans-diagnostic phenomenon linked to many psychological dimensions, in particular the impulsivity–hostility dimension. Many psychiatric disorders can lead people to behave aggressively, such as cluster B personality disorders [32], schizophrenia [33], intermittent explosive disorder [34], adjustment disorders with disturbance of conduct [35], manic states [16,36], substance use disorders [37,38], neurodevelopmental and cognitive disorders [39,40], and post-traumatic stress disorder (PTSD) [41].

Conversely, some psychiatric conditions are associated with reduced aggression (e.g., residual schizophrenia or prolonged and heavy cannabinoid use) or self-directed aggression such as depression, where aggression is typically directed toward the self, although suicide itself may carry significant hetero-aggressive implications [42,43,44].

Regarding self-injurious behaviors and suicidal behaviors (Non-Suicidal Self-Injury), both can be indicators of psychiatric vulnerability [45], with a significant overlap between these behaviors: approximately 50% of individuals engaging in self-harm attempt suicide at least once during their lifetime [46,47]. Self-injury is particularly prevalent during adolescence and early adulthood [13,48], with a prevalence of up to 70% among individuals with borderline personality disorder [49,50]; suicide represents the second leading cause of death among individuals aged 15–29 years [51] and is most commonly carried out by individuals with major depressive disorder [52], bipolar disorder, and schizophrenia [53]. Factors more strongly associated with suicide attempts include prior traumatic experiences, such as sexual abuse and victimization [54,55,56,57].

### 1.4. Aggressive Behavior Assessment Risk and Management

To prevent and safely manage aggressive and violent episodes in a hospital setting, it is necessary to establish a care plan that integrates clear management protocols with a comprehensive risk assessment from a multidisciplinary team [58]. In particular, for risk assessment, many questionnaires and scales are available for measuring aggressive behavior, albeit with differences in target settings and populations of application. Suicide risk assessment represents a continuous and dynamic process throughout the entire hospitalization period, taking into account clinically relevant phases during treatment and risk factors such as multiple psychiatric comorbidities, early trauma, social isolation, and substance use [57,59,60].

Aggressive behaviors are often preceded by non-violent agitation [61,62], the management of which represents a clinical and organizational priority to prevent escalation into overt aggression [63,64,65]. Management should follow a stepwise approach prioritizing non-pharmacological strategies [66], with pharmacological interventions reserved for cases where these prove ineffective.

De-escalation techniques are essential to prevent the worsening of critical situations [67]; post-event debriefing with both the patient and the clinical team is also important [68,69].

When de-escalation and preventive measures are ineffective, and risk increases, therapeutic strategies such as safety measures and targeted pharmacological treatment must be implemented. The main pharmacological classes used in the management of psychomotor agitation are benzodiazepines and antipsychotics, with preference given to those with sedative effects [70]. Among benzodiazepines, lorazepam—more effective when administered in combination with haloperidol [71]—and midazolam, which has a faster sedative effect than antipsychotics [72], are commonly used. The most frequently used antipsychotics include haloperidol [70,73], clotiapine [74], aripiprazole [73], and olanzapine [70]. If these treatments prove ineffective, ketamine may be administered in agreement with the anesthesiologist.

Physical restraint, mechanical constraint, seclusion and forced medication are extreme, restrictive interventions, implemented in cases of dangerous conditions of aggression and violence not otherwise manageable, which can lead to ethical dilemmas and psychological distress experienced by staff and patients, respectively, with possible harmful consequences for both [58].

The primary research aim of this study is to analyze self- and other-directed aggressive behaviors exhibited by patients hospitalized in an acute psychiatric ward over a one-year period. The secondary aims are to identify specific risk factors and describe prevention and management of aggressive behaviors in a hospital psychiatric setting.

## 2. Materials and Methods

### 2.1. Study Design, Setting, and Duration

This is a retrospective–prospective, observational, single-center study. The study duration was 12 months. Following ethical approval (3 December 2024), data were collected prospectively until 10 August 2025 and retrospectively until 11 August 2024. The study was conducted at the Service for Psychiatric Diagnosis and Care (SPDC) of AUSL-Modena in Baggiovara Civil Hospital (Modena, Italy). 

### 2.2. Sample Eligibility Criteria

The study included all hospitalizations under voluntary and involuntary treatment, according to Laws 180/78 and 833/78, occurring during the study period, of patients aged ≥ 14 years hospitalized in the SPDC of AUSL-Modena.

### 2.3. Assessment of Other-Directed Aggression Risk

To assess the risk of other-directed aggression, the Brøset Violence Checklist (BVC) [75] was applied. The scale was developed in Norway by Almvik and colleagues [75] and subsequently validated in Italy [76] and in several European psychiatric settings, demonstrating good predictive reliability for imminent violence [77,78], with a validity window of 24 h. High scores at admission are predictive of longer hospital stays [79].

The model is based on six indicators, including four behaviors (verbal threats, physical threats, attacks on objects and boisterousness) and two psychopathological features (confusion and irritability). Each indicator is scored dichotomously (1 = present; 0 = absent), with a maximum score of 6 (0: no risk; 1: low risk; 2–3: moderate risk; ≥4: high risk). A total score ≥ 2 indicates a high risk of aggressive behavior [80]. The assessment has a validity of 72 h for low or no risk, whereas for scores ≥ 2, more frequent reassessments are required, at each shift change and whenever clinically indicated. In our database, risk levels 2 and 3 (moderate risk) were merged, resulting in four categories: no risk (0), low (1), medium (2), and high (3).

### 2.4. Assessment of Suicide Risk

According to the Emilia Romagna Region Guidelines (ERRG) for the Prevention of Suicidal Behavior in Hospitals [81], 4 levels of suicide risk are defined and assigned at admission.

- Absent: No suicidal ideation; negative history; clinical condition not associated with suicide risk. Routine care and treatment are provided.
- Level 2—Low Risk: Presence of general risk factors, sporadic and low-intensity suicidal ideation; previous demonstrative attempts; positive family history; significant losses (emotional, economic, social); low-risk psychiatric disorders; moderate alcohol use. The following interventions are implemented: observation and empathic support by the clinical team; psychoeducation for patients and families; psychiatric evaluation if not already in a psychiatric setting.
- Level 3—Moderate Risk: Persistent but partially criticized suicidal ideation; recent predisposing factors; recent contact with healthcare services; severe psychiatric disorders (e.g., major depression, psychotic disorders, borderline personality disorder); substance or alcohol abuse; severe physical comorbidities. The preventive measures include previous measures and the following ones: active involvement of family members; individualized supervision and frequent monitoring; environmental safety checks; evaluation for transfer to a psychiatric unit; daily multidisciplinary monitoring; early linkage with community services.
- Level 4—High Risk: Persistent, intense, and uncritical suicidal ideation; recent suicide attempt; high-risk psychiatric and medical conditions; active substance abuse. For this risk level, continuous 24 h supervision and detailed post-discharge care planning are provided. Risk assessment must consider the combination and severity of these factors and requires continuous and dynamic monitoring during hospitalization [81].

Suicide risk assessment requires a dynamic, ongoing process, as risk may change according to the patient’s clinical condition.

### 2.5. Selected Sample Variables

The following variables were collected:

Self- and other-directed aggression risk, assessed using the ERRG for the Prevention of Suicidal Behavior in Hospitals and the BVC, respectively.

Socio-demographic characteristics: Sex, age, nationality, marital status, education level, housing and employment status, and parenthood.

Hospitalization variables: Clinical reason for hospitalization, voluntary and involuntary treatments, previous psychiatric hospitalizations, psychiatric diagnosis (ICD-9-CM) at discharge, length of stay (days), discharge destination, number and type of self- or other-directed aggressive behaviors during hospitalization, time of the day during which aggressive behavior occurred, preventive and management interventions, consequences of aggressive behaviors and Incident Reports (IR).

### 2.6. Study Procedure

The samples of hospitalizations and patients were divided into 4 subgroups based on the presence of self- and/or other-directed aggressive behavior:

1. No aggressive behavior.
2. Self-directed aggressive behavior.
3. Other-directed aggressive behavior.
4. Both self- and other-directed aggressive behavior.

The selected socio-demographic variables were analyzed in the sample of patients. The socio-demographic characteristics recorded at the first eligible hospitalization were used for patients with repeated admissions.

The hospitalization variables were analyzed in the sample of hospitalizations, comparing across these four subgroups of aggressive behavior. Because some hospitalizations were associated with the same patient, we accounted for within-patient correlation using a Generalized Estimating Equations (GEE) clustering model.

Additionally, the association between other-directed aggression risk, assessed through BVC, and suicide risk, evaluated by means of the ERRG for the Prevention of Suicidal Behavior in Hospitals, and the occurrence of self- and other-directed aggressive behaviors was evaluated.

Preventive and management interventions for aggressive behaviors, as well as their harmful consequences on patients and healthcare staff and the frequency of Incident (IR) in the study period, were also analyzed.

### 2.7. Statistical Analysis

Descriptive statistical analysis was conducted as follows:

- Mean ± standard deviation for continuous variables.
- Distribution analysis using the Shapiro–Wilk test.
- *t*-test for comparison of continuous variables that are normally distributed and Kruskal–Wallis test for non-normally distributed variables.
- Percentages, chi-square (χ^2^) test and Fisher’s exact test for categorical variables.

We analyzed AUC, sensitivity, specificity, positive predictive value, and negative predictive value of BVC to evaluate its validity in predicting short-term inpatient violence and aggression.

We analyzed socio-demographic variables at the patient level, whereas we analyzed clinical variables at the hospitalization level using Generalized Estimating Equations with robust variance clustered by patient.

We implemented one model of forward and backward stepwise multiple logistic regression with other-directed aggressive behavior (present = 1, absent = 0) as the dependent variable and selected variables as predictors.

Statistical significance was set at *p* < 0.05.

Data were analyzed using STATA version 19 (2025).

### 2.8. Ethical Considerations

This study was conducted in accordance with the Declaration of Helsinki. Data were collected through the computerized system used in SPDC, assigning each selected patient a progressive numerical code in order to anonymize the patient, and analyzed using statistical methods to obtain the information required for the research. Access to the data was granted to the study manager and collaborators, who were bound by the obligation of confidentiality and data processing in accordance with current regulations.

This study was approved on 3 December 2024 by the Ethics Committee of Area Vasta Emilia Nord (Protocol No. AOU 0034284/24) and received authorization from AUSL-Modena (Decision No. 20, dated 9 January 2025).

## 3. Results

We collected 411 hospitalizations corresponding to 370 unique patients. Thirty-two patients were readmitted, accounting for 41 hospitalizations beyond the first (27 patients had two hospital admissions, and five patients had more than two admissions).

In a total sample of 370 patients, we collected 108 (29.2%) patients who displayed aggressive behaviors: 11 (3%) showed self-directed aggression, 86 (23.2%) other-directed aggression, and 11 (3%) exhibited both aggressions during hospitalization.

### 3.1. Socio-Demographic Characteristics of Patient Sample (n = 370)

The total patient sample consisted of 177 men (47.8%) and 193 women (52.2%) with a mean age of 36.8 ± 16.2 years (SD), as reported in Table 1. Younger individuals were at greater risk of engaging in self-aggressive behaviors (mean age 20.55 ± 6.68 years, SD) or both types of aggression (mean age 18.33 ± 7.63 years, SD); conversely, the mean age of subjects with other-directed aggressive behaviors (mean = 36.13 ± 10.88 years, SD) was comparable to that of the overall sample. Age differed significantly across the four groups of subjects, highlighting that patients who manifested self-aggressive behaviors, and both self- and other-directed aggressive behaviors, were younger than others (chi2 = 40.27, *p* = 0.0001, Kruskal–Wallis test).

A total of 57 aggressive behaviors were recorded among men and 48 among women. Other-directed aggressive behaviors were more prevalent among males (*n* = 52, 62.7%) than females (*n* = 31, 37.3%) (Pearson’s Chi2 = 13.73; *p* = 0.003), whereas females more frequently exhibited self-aggressive behaviors (*n* = 9, 81.8%) or both self- and other-directed aggressive behaviors (*n* = 8, 72.7% vs. *n* = 3, 27.3%).

With regard to marital status, the frequency of aggressive behaviors, both self- and other-directed, was higher among unmarried people: 100% of subjects reporting self-aggressive behaviors or both types of aggression were single, whereas married or partnered individuals were the least likely to engage in aggressive behavior (Fisher’s exact; *p* = 0.014) (Table 1).

Regarding nationality, most patients were Italian (*n* = 274, 74.1%), followed by non-European nationals (*n* = 78, 21.1%) and European non-Italian nationals (*n* = 18, 4.9%) (Fisher’s exact; *p* = 0.020).

Most of the sample had no children (*n* = 278, 75.1% vs. *n* = 92, 24.9%); parenthood appeared to be a protective factor against self-aggressive behaviors, as none of the participants with children (*n* = 0, 0%) exhibited self-directed aggression (Fisher’s exact; *p* = 0.019).

A statistically significant difference was found between type of aggressive behavior and housing conditions (Fisher’s exact, *p* = 0.001); specifically, among subjects living with their family of origin (*n* = 157, 42.4%), self-aggressive behaviors were more frequent (*n* = 10, 90.9%).

Most of the sample had completed secondary school (*n* = 189, 51.1%) or lower secondary school (*n* = 134, 37.6%); 20 subjects (5.4%) had only completed primary school, and only 17 (4.6%) had obtained a university degree. Ten individuals (2.7%) required cultural mediation because of illiteracy and lack of knowledge of the Italian language; in this subgroup, aggressive behaviors were more frequent (SR ≥ 2; *p* < 0.05). Subjects who most frequently exhibited both self- and other-directed aggressive behaviors were those with lower secondary education (*n* = 10, 90.9%) (Fisher’s exact, *p* = 0.005).

Regarding employment status, we found statistically significant differences (Fisher’s exact, *p* < 0.001): students showed statistically significant rates of self-aggressive behaviors (*n* = 7, 63.6%) or both self- and hetero-aggressive behaviors (*n* = 8, 72.7%; SR ≥ 2, *p* < 0.05). Among the remaining employment categories, unemployment was significantly associated with hetero-aggressive behaviors (*n* = 43, 51.8%; SR ≥ 2; *p* < 0.05).

### 3.2. Clinical Characteristics of Hospitalization Sample (n = 411)

Regarding the patients hospitalized, 48.6% of them (*n* = 180) were admitted for their first psychiatric hospitalization, whereas 51.4% of them (*n* = 190) had previously been hospitalized in psychiatric settings. A significant prevalence of other-directed aggression was found among subjects involuntarily hospitalized (*n* = 67, 69.8%) compared with those voluntarily hospitalized, as shown in Table 2 (Fisher’s exact, *p* < 0.001).

The most frequent clinical reason for admission in the sample was acute psychosis (*n* = 160, 38.9%), which was also the condition most strongly associated with other-directed aggressive behaviors (*n* = 52, 54.2%) (Table 2).

The mean length of stay was significantly longer for individuals who engaged in self- (11± 6.85 days) or other-directed behaviors (18.21 ± 14.14 days) or in both aggressive behaviors (22.27 ± 13.75) compared with those who did not (8.72 ± 6.67 days) (Chi2 = 63.62, *p* = 0.0001, Kruskal–Wallis test).

The suicide risk levels evaluated at admission are reported in Table 3. Among patients hospitalized in the study period, 22 were admitted following suicidal ideation. High suicide risk was assigned to seven patients admitted following a suicide attempt by medication overdose and to eight patients admitted after a suicide attempt using violent methods, such as weapons.

**Figure 1:**
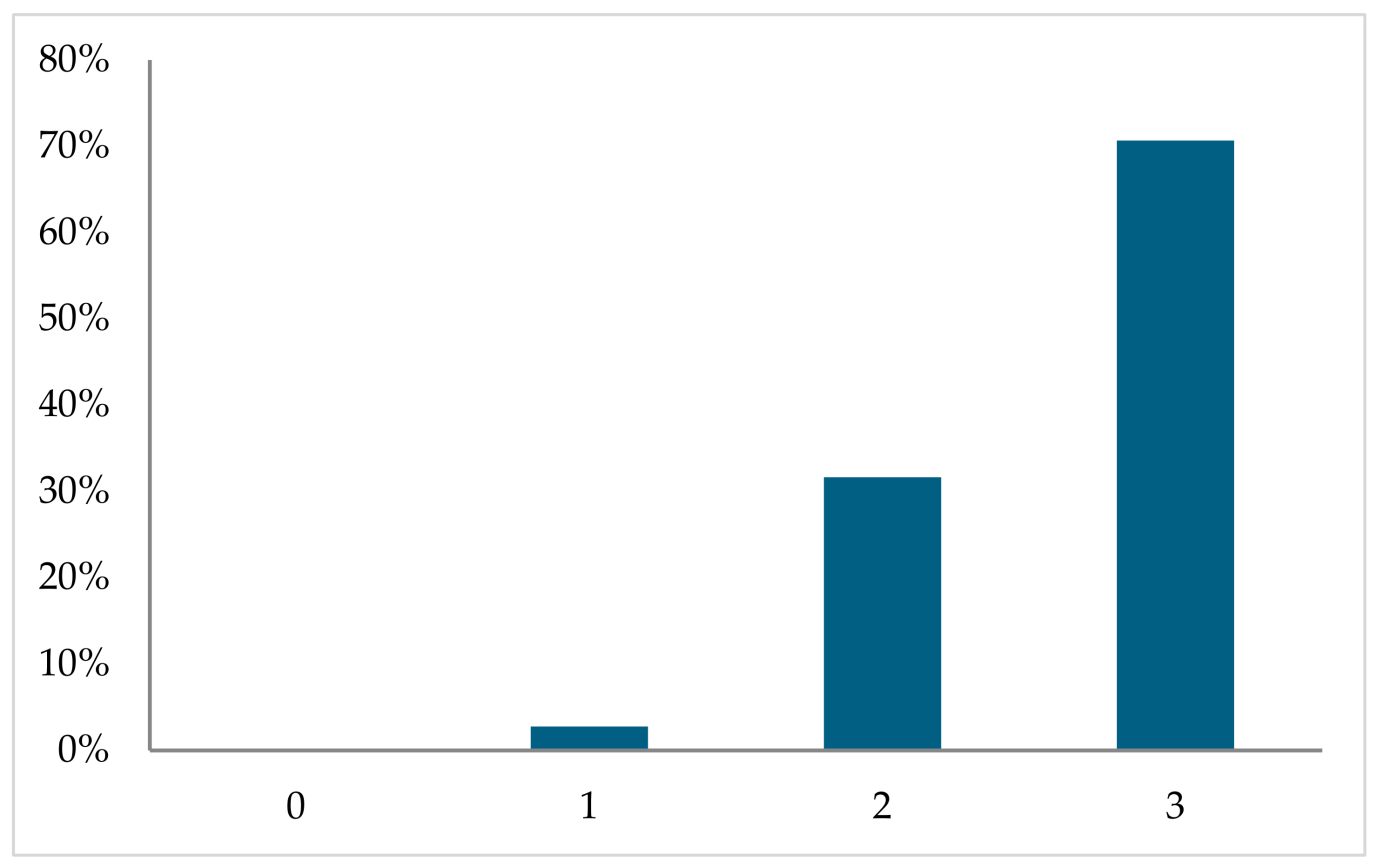
Observed other-directed aggression risk by BVC scale score.

With regard to aggression risk, as assessed by the BVC, 33 subjects (8.0%) were classified at admission as having no risk, 145 (35.3%) as low risk, 158 (38.4%) as medium risk, and 75 (18.2%) as high risk (Fisher’s exact, *p* < 0.001), with a significant correlation between medium or high aggression risk and the occurrence of hetero-aggressive behaviors (medium: *n* = 46, 47.9%; high: *n* = 46, 47.9%), as shown in Table 4. A positive correlation was also observed between high aggression risk and the presence of both self- and other-directed aggression (*n* = 7, 63.6%). We reported an observed risk for other-directed aggression of 31.6% at BVC = 2 and 70.7% at BVC = 3, as shown in Figure 1. We highlighted the following predictive performance metrics of the BVC scale for aggression: AUC = 0.841, sensitivity at BVC ≥ 2 = 96.3%, specificity at BVC ≥ 2 = 57.2%, positive predictive value = 44.2%, and negative predictive value = 97.8%.

Schizophrenia spectrum disorders (*n* = 59, 61.46%) and psycho-organic disorders were the diagnoses most strongly associated with other-directed aggressive behaviors (*n* = 3, 3.13%), whereas depressive disorders were often associated with self-aggression (*n* = 4, 36.36%), as reported in Table 5. Among conduct disorders, both self- and other-directed aggressive behaviors were significantly represented (*n* = 6, 54.56%).

Of the 370 patients examined, 145 (39.2%) had at least one organic comorbidity. Our analyses did not reveal any significant difference in the presence or absence of aggressive behaviors between patients with and without organic comorbidities.

It was also observed that 33.5% of hospitalized subjects (*n* = 124) showed substance abuse; however, no statistically significant difference in aggressive behaviors emerged between subjects with substance use and those without substance abuse.

No significant difference was found in discharge destination between subjects who did or did not exhibit self- or hetero-aggressive behaviors during hospitalization.

We used GEE clustering models to analyze clinical variables at the hospitalization level, as reported in Table 6. For self-directed aggression, we highlighted that each increase in the level of suicide risk is associated with an OR of 4.57 (95% CI: 2.40–8.68; *p* < 0.001); however, this result should be considered exploratory due to the small number of events (*n* = 22). For aggression directed toward others, we reported that each additional point on the BVC scale score is associated with an OR of 7.91 (95% CI: 5.13–12.20; *p* < 0.001) and previous hospitalization with an OR of 2.11, *p* = 0.015; however, the association loses significance when the analysis is limited to the first admission. The analysis based exclusively on the first admission substantially confirms the result regarding the BVC with an OR of 7.87 (95% CI: 4.85–12.76; *p* < 0.001).

### 3.3. Analysis of Self- and Other-Directed Aggressive Behavior and Safety Measures

In our sample, other-directed aggressive behaviors were divided into three categories, as shown in Table 7: verbal aggression (*n* = 133, 49.3%), physical aggression (*n* = 73, 27.0%), and aggression directed toward objects (*n* = 64, 23.7%). Most aggressive behaviors were carried out by subjects admitted under involuntary treatment, although no statistically significant difference was highlighted (*n* = 150, 55.6%; Pearson’s Chi2 = 0.7452; *p* = 0.689).

**Figure 2:**
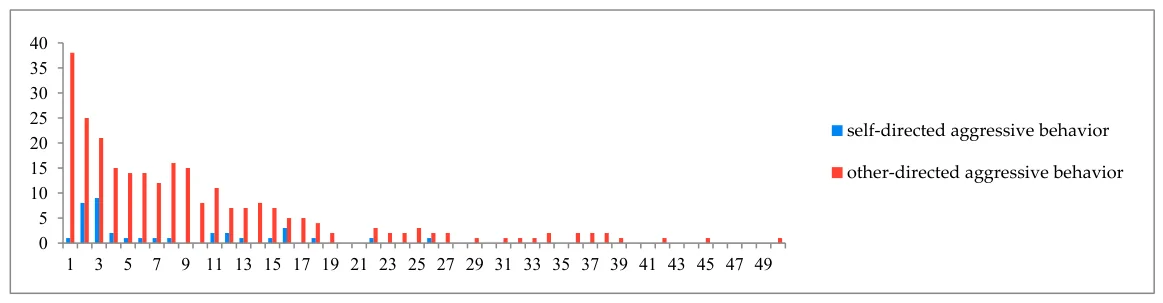
Self- and other-directed aggressive behavior per day of hospitalization.

Most aggressive behaviors occurred during the first days of hospitalization, with a peak on day 1 (*n* = 38, 14.1%) for other-directed aggression and between day 2 (*n* = 8, 21.6%) and day 3 (*n* = 9, 24.3%) for self-directed aggression (Figure 2).

Most aggressive behaviors occurred during the afternoon shift (*n* = 125, 46.3%), followed by the morning shift (*n* = 114, 42.2%), whereas violent episodes during the night shift were markedly less frequent (*n* = 31, 11.5%) (Pearson’s Chi2 = 9.8303, *p* = 0.043). Aggression toward objects occurred during the afternoon shift in 62.5% of cases (*n* = 40) (SR ≥ 2, *p* < 0.05).

In only 13.3% of cases (*n* = 36) medication was not required after aggressive episodes, predominantly in cases of verbal aggression (*n* = 23, 63.9%); in the vast majority of cases, however, medication was administered orally (*n* = 114, 42.2%) or intramuscularly (*n* = 120, 44.4%) (Pearson’s Chi2 = 16.08, *p* = 0.003). In cases of verbal aggression, oral medication was more common (*n* = 66, 49.6%), whereas in cases of physical aggression, intramuscular medication predominated (*n* = 42, 57.5%).

In most cases, relational containment by healthcare professionals was sufficient (*n* = 141, 52.2%), especially in cases of verbal aggression (*n* = 93, 69.9%), whereas in other cases, the intervention of security personnel was required (*n* = 129, 47.8%), particularly when aggression was directed toward people (*n* = 50, 68.5%) or objects (*n* = 39, 60.9%) (Pearson’s Chi2 = 33.70, *p* < 0.001). Mechanical restraint was never used in accordance with local guidelines.

With respect to harmful consequences, no damage of any kind was reported in 85.9% of cases (*n* = 232), whereas some cases of physical aggression resulted in harm to patients (*n* = 22, 8.1%), healthcare workers (*n* = 6, 2.2%), or both (*n* = 2, 0.7%). Structural damage occurred in only 3.0% of cases (*n* = 8), most of which consisted of aggressive acts directed at objects (*n* = 6, 9.4%) (Fisher’s exact, *p* < 0.001).

Finally, we examined the frequency of Incident Reports. In most cases, an Incident Report was not required (*n* = 36, 13.3%), especially in cases of verbal violence, for which it was filed in only six cases (4.5%). By contrast, Incident Reports were required mainly in cases of aggression directed toward people (*n* = 19, 26.0%; Pearson’s Chi2 = 19.96, *p* < 0.001).

Regarding the pharmacological treatment of self-directed aggressive behavior, we highlighted a statistically significant difference across the three different modalities (Fisher’s exact, *p* = 0.002), with more prevalent use of oral treatment in cases of suicidal ideation and injectable drugs in cases of suicide attempts with violent methods (Table 3).

When considering the non-pharmacological containment adopted in cases of self-aggression, in the vast majority of cases, relational containment was sufficient (*n* = 34, 91.9%), and only in 8.1% of cases (*n* = 3) was intervention by the hospital’s internal security required, with no significant difference between episodes of suicidal ideation, drug ingestion and suicide attempts through violent methods, as reported in Table 3.

### 3.4. Multiple Logistic Regression

We performed one forward and backward stepwise multiple logistic regression analysis using other-directed aggressive behaviors as the dependent variable and selected variables as predictors. A statistically significant association emerged between non-European nationality and the presence of other-directed aggressive behavior (OR = 1.966, 95% IC: 0.03–0.91, *p* = 0.023).

## 4. Discussion

Our study analyzed self- and other-directed aggressive behaviors among patients hospitalized in an acute psychiatric ward, the SPDC of AUSL-Modena, over a one-year period.

### 4.1. Socio-Demographic Characteristics and Self- and Other-Directed Aggressive Behaviors

A higher prevalence of aggressive behavior emerged among male patients, particularly with regard to other-directed aggressive behavior. This finding is consistent with the literature, which shows that physical aggression is more frequently expressed by males, whereas indirect or verbal aggression is substantially comparable between the two sexes [82]. Conversely, according to the literature, self-injurious behaviors and suicidal threats are more prevalent among females, plausibly due to generally higher levels of depression and greater emotional dysregulation, with a frequency approximately twice that observed in males [83]. This pattern was also supported by one of our multiple logistic regressions. In our study, the difference between the two sexes was also significant with respect to age, especially for self-injurious behaviors, which were more frequent among younger women, as confirmed by previous studies [13,48,84]. The mean age of all subjects hospitalized during the observation period was 36.8 years, which was comparable to that of subjects with other-directed aggressive behaviors (36.1 years), whereas the mean age of subjects with self-aggressive behaviors was lower (20.6 years), as well as that of subjects showing both self- and other-directed aggression, who were even younger (18.3 years). These findings are in line with the literature [13,85]. Other studies emphasize that, during adolescence, the brain is still undergoing maturation, particularly in the prefrontal cortex, which is responsible for impulse control, planning, and evaluation of consequences [86]. By contrast, the amygdala, which regulates emotions and reward processing, develops earlier, creating an imbalance between impulsivity and inhibitory capacity [87,88,89].

With regard to marital status, we found an increase in self-injurious behaviors among unmarried people, likely due both to their younger age and to a reduced ability to form meaningful social relationships, a finding fully consistent with the literature. A 2023 study showed that loneliness and social exclusion may elicit aggressive reactions and reduce the ability to inhibit them [90]. The results regarding housing conditions also showed greater aggression among adolescents: subjects who displayed greater aggression, both self- and other-directed, were those living with their family of origin, namely those with lower levels of autonomy and individual coping capacity, as supported by the literature [91].

Having children appeared to be a protective factor against aggression: subjects with offspring did not engage in self-aggressive behaviors, and only some of them showed other-directed aggressive behaviors. Population studies have shown that parenthood is associated with a lower risk of suicide [92,93], whereas the risk of self-harm appears to be more strongly influenced by the quality of family relationships than by parenthood itself.

We also found significant differences with respect to educational level: in most cases, self-aggressive behaviors were observed in students and in subjects with lower secondary education. On the one hand, this suggests that a higher educational level may act as a protective factor against self-aggressive behavior, as supported by the literature [94,95]; on the other hand, it further underscores the young age of subjects exhibiting self-injurious behaviors [96].

Subjects of non-European nationality more frequently displayed aggressive behaviors, particularly other-directed aggression. Some studies have examined the association between aggressive behaviors in adolescents and ethnicity [97], reporting a higher incidence among African American adolescents. However, the same study also emphasized the role of situational, individual, and demographic risk factors, such as alcohol or substance use, friendships involving violent behavior, low educational level, limited family supervision, depression, family conflict, and disadvantaged socioeconomic conditions. It is also important to consider the history of non-European subjects included in our sample, who were often migrants and victims of abuse and violence during migration journeys, in accordance with another study that found high rates of traumatic experiences and abuse among migrant adolescents [98].

### 4.2. Clinical Characteristics and Self- and Other-Directed Aggressive Behaviors

A statistically significant difference emerged in the number of hetero-aggressive behaviors, which were more frequent among subjects involuntarily hospitalized. This result confirms the literature, which reports that approximately one in five patients admitted involuntarily to an acute psychiatric ward commits at least one violent act [26]. Other-directed aggressive behavior also appears to be a significant factor associated with longer hospitalization, reflecting the greater severity and complexity of clinical cases requiring prolonged clinical and rehabilitative interventions to promote adequate treatment adherence, as previously reported [99]. Our data also confirm the literature regarding reasons for admission: subjects hospitalized for acute psychosis frequently showed aggressive behaviors significantly [100,101], whereas self-injurious behaviors were more frequent in patients admitted following a suicide attempt or because of self-harm risk [102]. Finally, subjects most likely display both self- and other-directed aggressive behaviors when admitted due to intoxication with substances, in agreement with the literature [37,103].

Regarding discharge diagnosis, the diagnosis most strongly associated with other-directed aggression was schizophrenia spectrum disorder, in line with the literature [33], followed by psycho-organic disorders, thereby confirming previous findings highlighting aggression particularly in the presence of frontal lobe lesions [104]. Although most individuals with schizophrenia do not exhibit violent behavior, some evidence suggests that the tendency toward violence or aggression is, on average, higher in this population than in the general population [19,105], especially when there is a long duration of untreated psychosis [106,107]. Comorbid schizophrenia and substance abuse represent a condition consistently associated with increased aggressive behaviors and higher crime rates [19].

Major depressive disorder was frequently associated with the absence of aggressive behaviors; when aggression was present, it was generally self-directed. The literature confirms depression as a major risk factor for suicidal behavior, for which self-injury may represent a precursor [52,108,109,110,111,112]. Adolescents diagnosed with conduct disorder were the most likely to exhibit both self- and other-directed aggressive behaviors, fully in line with the literature [110,113,114,115]. A 2022 study on adolescents with conduct disorder associated callous–unemotional traits with a reduced ability to experience guilt or remorse, lack of empathy, and absence of fear of consequences, whereas irritability and aggression appeared to be associated with dominance-related interpersonal traits [116].

### 4.3. Assessment and Management of Self- and Other-Directed Aggressive Behaviors

The highest risk for suicide at admission according to the Emilia-Romagna regional guidelines was significantly attributed to subjects admitted following a suicide attempt using violent methods. This is consistent with the literature: a 2017 study found a completed suicide rate 37 times higher than that of the general population among individuals who had engaged in non-fatal self-harm in the previous 12 months [117]. This risk was particularly high among subjects who had used violent methods, especially firearms [118,119]. Suicide risk was concentrated especially in the first 30 days after the initial self-harm act [117], underscoring the importance of accurate assessment and intensive monitoring during the early stages of hospitalization [120].

Our results also showed the good predictive validity of the Brøset Violence Checklist (BVC), with a statistically significant prevalence of other-directed aggressive behaviors among subjects scoring two or higher on the scale. BVC shows good discrimination. The high sensitivity and negative predictive value support use as a rule-out screening tool. Specificity and positive predictive value are moderate; a positive BVC result should not be treated as proof that aggression will occur. This finding confirms the previous literature on the predictive value of the BVC, which appears to reduce the use of coercive measures and aggression-related incidents [76,78,80].

Other-directed aggression occurred predominantly in verbal form, followed by physical aggression and finally aggression against objects, in accordance with the literature [121]. We then analyzed the relationship between aggressive behaviors and the day of hospitalization on which they occurred, showing a different distribution between self-directed acts, which occurred mostly on the second and third days of hospitalization, and other-directed acts, which were recorded predominantly on the first day of admission, as confirmed by previous studies [122], suggesting both the severity of the clinical conditions and a paradoxical worsening due to a sudden and dramatic awareness of the disease by the patient at admission [26].

With regard to the time of day of aggressive behaviors, most of them occurred during the afternoon shift, especially aggression episodes directed at objects or ward structures, plausibly because of reduced ward activity and lower staff presence. This result is in line with the literature [11,123,124]. In cases of aggressive episodes occurring during the night shift, other studies suggest that an altered state of consciousness, such as a state of confusion, may induce more frequent aggressive behaviors at night [125,126].

For the management of aggressive episodes, medication was required in 86.6% of violent incidents. In cases of verbal aggression, oral administration was often sufficient, whereas in episodes of physical aggression, intramuscular administration was frequently required, similarly to what has been reported in the literature [63,127,128].

Regarding non-pharmacological containment, in 52.2% of cases, a relational intervention by healthcare staff was sufficient, particularly de-escalation strategies and emotional validation, especially in cases of verbal aggression, as recommended in the literature [67]. Several studies have confirmed the high frequency of aggressive incidents in psychiatric settings and their associated harmful consequences [24,26,129,130]. Intervention by hospital security staff, who are present on the ward 24 h a day, was required in 8.1% of self-aggressive episodes and in 47.8% of other-directed aggressive episodes, especially those directed at people or objects. This type of intervention proved effective in containing other-directed aggressive behaviors and made it possible to avoid the use of mechanical restraint in 100% of cases, even in episodes of extreme violence. This measure has not been used on the ward since 2021, in accordance with departmental guidelines favoring a non-coercive, more protective, and ethically grounded approach to patient care. Indeed, mechanical restraint may have significant physical and psychological consequences, including the onset of post-traumatic symptoms [131]. Isolation measures, such as confining an aggressive patient in a seclusion room, not in use in the SPDC ward, although often perceived as less invasive than mechanical restraint, may have comparable adverse effects [132,133]. The effectiveness of relational containment is also supported by the international literature [134]. A study conducted in 2015 proposed the “Safewards Model”, consisting of simple relational interventions aimed at improving communication and ward climate, and found it effective in significantly reducing both conflict episodes and the use of containment measures [135].

With regard to the consequences of other-directed aggressive episodes recorded in our study, physical aggression resulted in harmful consequences for patients in 8.1% of episodes, for staff in 2.2%, and for both in 0.7% of cases. Episodes involving structural or object damage (breakage or damage to property) were more frequent, reaching 9.4%.

In 13.3% of cases, a formal report was filed through the Incident Report (IR) procedure, mainly in relation to acts directed toward other persons (26%), in line with the literature [136,137,138]. The use of the Incident Reporting system reflects a ward culture based on risk management through the identification of all harmful or potentially dangerous situations, with the aim of ensuring optimal care and patient safety, identifying system errors, and preventing future harm.

### 4.4. Study Limitations and Advantages

This study has several limitations, particularly the small sample size of subgroups; the limited follow-up duration; and the naturalistic, retrospective–prospective and single-center design, which prevent us from generalizing the findings. However, one advantage of the study could be the direct observation and analysis of aggressive behavior in a real-world setting, in this case, an acute psychiatric ward, thereby providing detailed insights into this complex healthcare issue, which carries significant clinical and ethical implications.

## 5. Conclusions

This study analyzed episodes of self- and other-directed aggression recorded over one year in the SPDC of AUSL-Modena, identifying their main predictive factors and management strategies. Other-directed aggressive behaviors were more frequent among male patients, whereas self-injurious behaviors and suicidal ideation were more prevalent among females. Adolescents represented the population at highest risk of aggressive behavior, with a frequent co-occurrence of self- and other-directed aggression, likely related to greater impulsivity and incomplete maturation of executive functions. Non-European nationality was also associated with an increased risk of violence, probably in relation to social and trauma-related vulnerability factors. This finding highlights the need for greater clinical and social attention toward young migrants, who may be particularly vulnerable and at risk of developing behavioral difficulties.

From a diagnostic perspective, major depressive disorder was mainly associated with suicidal and self-injurious behaviors, whereas other-directed aggression was more frequent among subjects with schizophrenia spectrum disorders. However, aggression should not be regarded as a stable trait of psychiatric illness, but rather as an expression of impaired impulse control and disrupted contact with reality, making systematic and dynamic risk assessment during hospitalization essential.

The assignment of suicide risk levels and other-directed aggression risk through the use of the Brøset Violence Checklist allows for early identification of warning signs, thereby reducing the severity of aggressive episodes and the need for coercive measures. The use of verbal de-escalation strategies and pharmacological containment supplemented, when necessary, by security staff intervention, represents a safer and more respectful approach than mechanical restraint.

To safely prevent and manage episodes of aggression and violence in a hospital setting, it is necessary to define a care plan that integrates clear management protocols with a comprehensive risk assessment conducted by a multidisciplinary team. A structured approach to risk management, combined with personalized rehabilitative interventions and continuity of care, can help improve both the safety of the therapeutic environment and clinical outcomes. Supervision, space management, the ability to recognize signs of change through accurate assessment, the establishment of a trusting relationship, and appropriate pharmacological treatment are essential tools for ensuring patient safety and providing support during the most critical moments.

## Figures and Tables

**Table 1:** Socio-demographic characteristics of patients admitted to the SPDC, stratified by type of aggressive behavior.

Variables	No Aggressive Behavior *n* (%)	Self-Directed Aggressive Behavior *n* (%)	Other-Directed Aggressive Behavior *n* (%)	Both Self- and Other-Directed Aggressive Behavior *n* (%)	Statistical Test Probability
Sex, *n* (%)					Pearson’s Chi2 = 13.73; *p* = 0.003
Male	120 (45.3%)	2 (18.2%)	52 (62.7%) *	3 (27.3%)	
Female	145 (54.7%)	9 (81.8%)	31 (37.3%)	8 (72.7%)	
Age					Chi2 = 40.27, *p* = 0.0001, Kruskal–Wallis test
Years, (m ± SD)	39.92 ± 14.88	20.55 ± 6.68	36.13 ± 10.88	18.33 ± 7.63	
Marital Status, *n* (%)					Fisher’s exact test; *p* = 0.014
Single	164 (61.9%)	11 (100%) *	61 (73.5%)	11 (100%) *	
Married/Cohabiting	81 (30.6%) *	0 (0%)	16 (19.3%)	0 (0%)	
Divorced/Separated/Widowed	20 (7.5%)	0 (0%)	6 (7.2%)	0 (0%)	
Nationality, *n* (%)					Fisher’s exact test; *p* = 0.020
Italian	205 (77.4%) *	9 (9%)	50 (60.2%)	10 (90.9%)	
Non-Italian European	15 (5.7%)	0 (0%)	3 (3.6%)	0 (0%)	
Non-European	45 (17%)	2 (18.2%)	30 (36.1%) *	1 (9.1%)	
Parenthood, *n* (%)					Fisher’s exact test; *p* = 0.019
With children	74 (27.9%) *	0 (0%)	18 (21.7%)	0 (0%)	
Without children	191 (71.1%)	11 (100%)	65 (78.3%)	11 (100%)	
Living Conditions, *n* (%)					Fisher’s exact test; *p* = 0.001
Acquired family	64 (24.2%)	0 (0%)	15 (18.1%)	1 (9.1%)	
Family of origin	110 (41.5%)	10 (90.9%) *	30 (36.1%)	7 (63.6%)	
Living alone	59 (22.3%)	0 (0%)	16 (19.3%)	0 (0%)	
Other	32 (12.1%)	1 (9.1%)	22 (22.9%) *	3 (27.3%)	
Education Level, *n* (%)					Fisher’s exact test; *p* = 0.005
Primary school	13 (4.9%)	0 (0%)	7 (8.4%)	0 (0%)	
Lower secondary	94 (35.5%)	6 (54.5%)	24 (28.9%)	10 (90.9%) *	
Upper secondary	141 (53.2%)	5 (45.5%)	42 (50.6%)	1 (9.1%)	
University degree	14 (5.3%)	0 (0%)	3 (3.6%)	0 (0%)	
Illiterate	3 (1.1%)	0 (0%)	7 (8.4%) *	0 (0%)	
Employment Status, *n* (%)					Fisher’s exact test; *p* < 0.001
Employed	85 (32.1%)	1 (9.1%)	22 (26.5%)	0 (0%)	
Unemployed	100 (37.7%)	3 (27.3%)	43 (51.8%) *	3 (27.3%)	
Pension	34 (12.8%)	0 (0%)	11 (13.2%)	0 (0%)	
Student	46 (17.4%)	7 (63.6%)	7 (8.4%)	8 (72.7%)	

Notes: * *p* < 0.05 (standardized residuals ≥ 2).

**Table 2:** Clinical characteristics of the sample stratified by type of aggressive behavior.

Variables	No Aggressive Behavior	Self-Directed Aggressive Behavior	Other-Directed Aggressive Behavior	Both Self- and Other-Directed Aggressive Behavior	Statistical Test Probability
Clinical Reason for Admission, *n* (%)					Fisher’s exact test, *p* < 0.001
Acute psychosis	106 (36.2%)	2 (18.2%)	52 (54.2%)	0 (0%)	
Agitation/excitement state	90 (29.7%)	0 (0%)	27 (28.1%)	6 (50.0%)	
Self-harm/suicide risk	69 (23.5%)	8 (72.7%)	9 (9.4%)	2 (18.2%)	
Others	38 (12.5%)	1 (9.1%)	8 (8.3%)	4 (36.4%)	
Admission Type, *n* (%)					Fisher’s exact test, *p* < 0.001
Involuntary admission	119 (40.6%)	4 (36.4%)	67 (69.8%)	1 (9.1%)	
Voluntary admission	174 (59.4%)	7 (63.6%)	29 (30.2%)	10 (90.9%)	
Hospitalization duration (m ± SD)					
Days	8.72 ± 6.67	11 ± 6.85	18.22 ± 14.14	22.27 ± 13.75	Chi2 = 63.62, *p* = 0.0001, Kruskal–Wallis test

**Table 3:** Suicide risk assessment and management.

	Suicidal Ideation *n* (%)	Drug Ingestion *n* (%)	Suicide Attempt Through Violent Methods *n* (%)	
Low risk (Level 1)	2 (9.1%)	0 (0%)	0 (0%)	
Moderate risk (Level 2)	13 (59.1%)	0 (0%)	0 (0%)	
High risk (Level 3)	7 (31.8%)	7 (100%)	8 (100%)	
Pharmacological treatment of suicide risk				
No pharmacological treatment	4 (18.18%)	2 (28.57%)	0 (0%)	Fisher’s exact *p* = 0.002
Oral pharmacological treatment	15 (68.18%)	4 (57.14%)	1 (12.5%)	
Intramuscular (IM) pharmacological treatment	3 (13.64%)	1 (14.29%)	7 (87.5%)	
Non-pharmacological containment of suicide risk				Fisher’s exact *p* = 0.356
Relational interventions	21 (95.45%)	6 (85.71%)	7 (25%)	
Intervention of the hospital’s internal security	1 (4.55%)	1 (14.29%)	1 (12.50%)	

**Table 4:** Risk of aggression at admission assessed using the Brøset Violence Checklist (BVC), stratified by type of aggressive behavior.

BVC Score at Admission	No Aggressive Behavior *n* (%)	Self-Directed Aggressive Behavior *n* (%)	Other-Directed Aggressive Behavior *n* (%)	Both Self- and Other-Directed Aggressive Behavior *n* (%)	Observed Risk	Wilson 95% IC
0 (absent)	31 (10.6%)	2 (18.2%)	0 (0.0%)	0 (0.0%)	0.0%	0.0–10.4%
1 (low)	135 (46.1%)	6 (54.5%)	4 (4.2%)	0 (0.0%)	2.8%	1.1–6.9%
2 (medium)	106 (36.2%)	2 (18.2%)	46 (47.9%)	4 (36.4%)	31.6%	24.9–39.3%
3 (high)	21 (7.2%)	1 (9.1%)	46 (47.9%)	7 (63.6%)	70.7%	59.6–79.8%

**Table 5:** Psychiatric diagnoses (ICD-9-CM) of patients discharged from the SPDC, stratified by type of aggressive behavior.

ICD-9-CM Psychiatric Diagnosis	No Aggressive Behavior *n* (%)	Self-Directed Aggressive Behavior *n* (%)	Other-Directed Aggressive Behavior *n* (%)	Both Self- and Other-Directed Aggressive Behavior *n* (%)
Psycho-organic disorders	1 (0.34%)	0 (0%)	3 (3.13%)	0 (0%)
Schizophrenia spectrum disorders	131 (44.71%)	2 (18.18%)	59 (61.46%)	1 (9.09%)
Bipolar disorder—manic episode	38 (12.97%)	0 (0%)	11 (11.46%)	0 (0%)
Depressive disorders	38 (12.97%)	4 (36.36%)	1 (1.04%)	2 (18.18%)
Personality disorders	52 (17.75%)	2 (18.18%)	14 (14.58%)	2 (18.18%)
Anxiety disorders	5 (1.71%)	1 (9.09%)	0 (0%)	0 (0%)
Substance use disorders	3 (1.02%)	0 (0%)	2 (2.08%)	0 (0%)
Intellectual disability/autism	6 (2.05%)	0 (0%)	1 (1.04%)	0 (0%)
Conduct disorders	19 (6.48%)	2 (18.18%)	5 (5.21%)	6 (54.56%)

**Table 6:** GEE hospitalization-level analyses.

Models and Variables	OR	95% CI	*p*
Self-directed aggression GEE admission risk ^1^			
Suicide risk score	4.57	[2.40. 8.68]	<0.001
Self-directed aggression first-admission logit admission risk ^2^			
Suicide risk score	4.29	[2.16. 8.51]	<0.001
Other-directed aggression GEE admission model ^3^			
BVC score	7.91	[5.13. 12.20]	<0.001
Involuntary treatment	1.29	[0.72. 2.32]	0.393
Previous hospitalizations	2.11	[1.16. 3.86]	0.015
Substance use	0.71	[0.38. 1.32]	0.273
Other-directed aggression first-admission logit admission model ^4^			
BVC score	7.89	[4.87. 12.78]	<0.001
Involuntary treatment	1.46	[0.78. 2.71]	0.236
Prior hospitalizations	1.60	[0.87. 2.95]	0.134
Substance use	0.67	[0.35. 1.26]	0.216
Other-directed aggression GEE clinical sensitivity ^3^			
BVC score	7.87	[5.10. 12.16]	<0.001
Involuntary treatment	1.22	[0.66. 2.24]	0.530
Prior hospitalizations	2.14	[1.17. 3.91]	0.013
Substance use	0.72	[0.38. 1.36]	0.308
Schizophrenia spectrum disorders	1.22	[0.67. 2.23]	0.512
Other-directed aggression GEE first-admission logit clinical sensitivity ^3^			
BVC score	7.87	[4.85. 12.76]	<0.001
Involuntary treatment	1.40	[0.74. 2.67]	0.300
Prior hospitalizations	1.62	[0.87. 2.99]	0.126
Substance use	0.68	[0.36. 1.29]	0.236
Schizophrenia spectrum disorders	1.15	[0.61. 2.15]	0.669

^1^: N = 411, events = 22, patients = 370; ^2^: N = 370, events = 20, patients = 370; ^3^: N = 409, events = 106, patients = 368; ^4^: N = 368, events = 86, patients = 368.

**Table 7:** Other-directed aggressive behavior characteristics and management.

Variables	Verbal Aggression *n* (%)	Physical Aggression *n* (%)	Aggression Against Objects *n* (%)	Statistical Test Probability
Working Shift				Pearson’s Chi2 = 9.83, *p* = 0.043
Morning	62 (46.6%)	34 (46.6%)	18 (28.1%)	
Afternoon	53 (39.8%)	32 (43.8%)	40 (62.5%) *	
Night	18 (13.5%)	7 (9.6%)	6 (9.3%)	
Pharmacological Treatment				Pearson’s Chi2 = 16.08, *p* = 0.003
None	23 (17.3%)	9 (12.3%)	4 (6.25%)	
Oral (OS)	66 (49.6%) *	22 (16.5%)	26 (40.6%)	
Intramuscular (IM)	44 (33.1%)	42 (57.5%) *	34 (53.1%)	
Non-pharmacological Containment				Pearson’s Chi2 = 33.70, *p* < 0.001
Relational approach	93 (69.9%) *	23 (31.5%)	25 (39.1%)	
Enhanced observation	40 (30.1%)	50 (68.5%) *	39 (60.9%) *	
Admission Status				Pearson’s Chi2 = 0.74, *p* = 0.689
Involuntary	74 (55.6%)	43 (58.9%)	33 (51.6%)	
Voluntary	59 (44.3%)	30 (41.1%)	31 (48.4%)	
Harmful Consequences				Fisher’s exact, *p* < 0.001
None	127 (95.4%)	54 (74.0%)	51 (79.7%)	
Patient	5 (6.8%)	11 (15.1%) *	6 (9.4%)	
Healthcare worker	0 (0.0%)	5 (6.8%) *	1 (1.6%)	
Patient and healthcare worker	0 (0.0%)	2 (2.7%) *	0 (0.0%)	
Objects/structure	1 (0.8%)	1 (1.4%)	6 (9.4%) *	
Incident Report				Pearson’s Chi2 = 19.96, *p* < 0.001
Absent	127 (95.5%) *	54 (74.0%)	53 (82.8%)	
Present	6 (4.5%)	19 (26.0%) *	11 (17.2%)	

Notes: * *p* < 0.05 (standardized residuals ≥ 2).

## Data Availability

The datasets presented in this article are not readily available due to privacy restrictions. Requests to access the datasets should be directed to the corresponding author.

## Abbreviations

The following abbreviations are used in this manuscript:

SPDC	Service for Psychiatric Diagnosis and Care
BVC	Brøset Violence Checklist
ERRG	Emilia-Romagna Region Guidelines
ICD-9-CM	International Classification of Diseases, 9th Revision—Clinical Modification
